# Multi-channel framelet denoising of diffusion-weighted images

**DOI:** 10.1371/journal.pone.0211621

**Published:** 2019-02-06

**Authors:** Geng Chen, Jian Zhang, Yong Zhang, Bin Dong, Dinggang Shen, Pew-Thian Yap

**Affiliations:** 1 Department of Radiology and Biomedical Research Imaging Center (BRIC) University of North Carolina, Chapel Hill, United States of America; 2 School of Information and Electrical Engineering, Hunan University of Science & Technology, Xiangtan, China; 3 Vancouver Research Center, Huawei, Burnaby, Canada; 4 Beijing International Center for Mathematical Research, Peking University, Beijing, China; 5 Department of Brain and Cognitive Engineering, Korea University, Seoul, Korea; Center for Neuroscience and Regenerative Medicine, UNITED STATES

## Abstract

Diffusion MRI derives its contrast from MR signal attenuation induced by the movement of water molecules in microstructural environments. Associated with the signal attenuation is the reduction of signal-to-noise ratio (SNR). Methods based on total variation (TV) have shown superior performance in image noise reduction. However, TV denoising can result in stair-casing effects due to the inherent piecewise-constant assumption. In this paper, we propose a tight wavelet frame based approach for edge-preserving denoising of diffusion-weighted (DW) images. Specifically, we employ the unitary extension principle (UEP) to generate frames that are discrete analogues to differential operators of various orders, which will help avoid stair-casing effects. Instead of denoising each DW image separately, we collaboratively denoise groups of DW images acquired with adjacent gradient directions. In addition, we introduce a very efficient method for solving an *ℓ*_0_ denoising problem that involves only thresholding and solving a trivial inverse problem. We demonstrate the effectiveness of our method qualitatively and quantitatively using synthetic and real data.

## Introduction

Diffusion MRI affords in vivo insights into brain tissue microstructure and allows reconstruction of white matter pathways for neuroscience studies involving development, aging, and disorders [1–5]. However, since diffusion MRI derives its contrast from MR signal attenuation, it suffers from low signal-to-noise-ratio (SNR), which complicates subsequent quantitative analyses. To improve SNR, multiple repetitive scans are typically acquired and averaged for noise reduction. This however inevitably prolongs acquisition times and is hence prohibitive in clinical settings. Post-acquisition algorithms, such as total variation (TV) denoising [6], have been widely adopted due to their ability to remove noise without requiring additional acquisition time.

**Fig 1:**
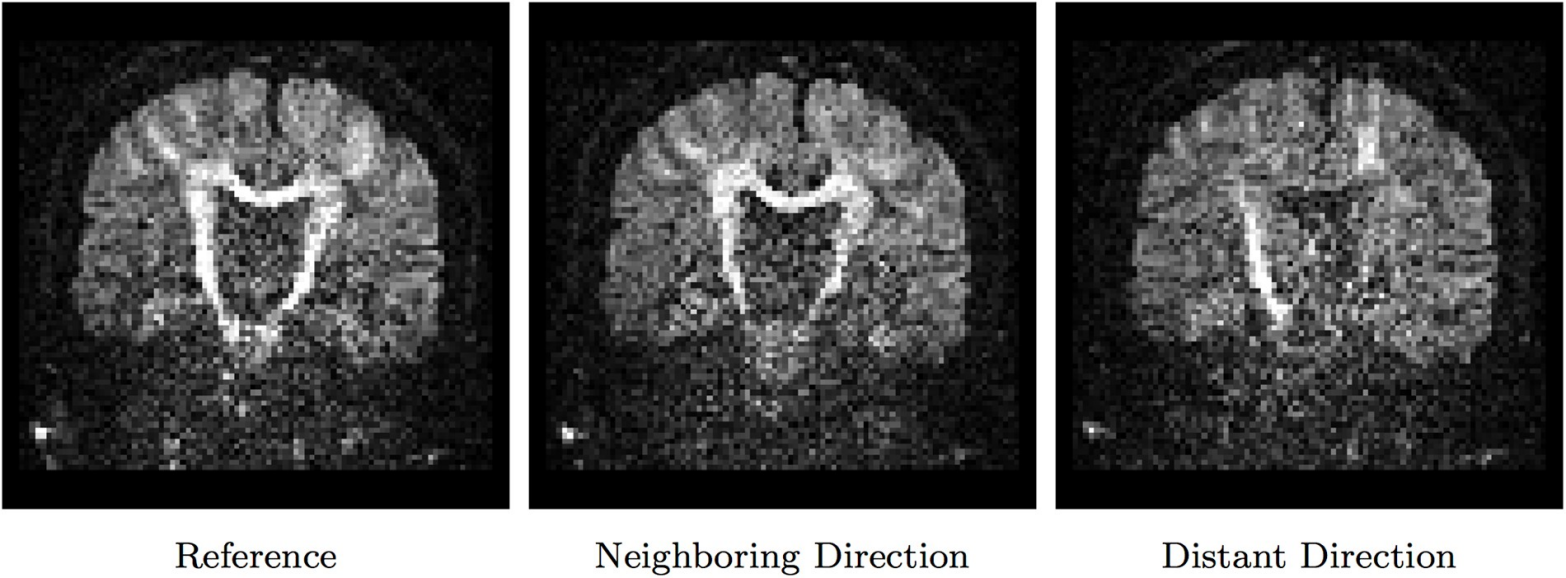
Diffusion-weighted images scanned at different gradient directions. The left and middle images were scanned with similar gradient directions. The right image was scanned at a nearly perpendicular gradient direction with respect to the reference.

Diffusion-weighted (DW) images are typically acquired with non-collinear gradient directions. As shown in Fig 1, DW images that are scanned with similar gradient directions share a lot of commonalities. However, these commonalities diminish very quickly if the difference between the gradient directions increases. Denoising performance can be improved by making full use of information between images scanned with similar gradient directions; however, images scanned with very different gradient directions have to be avoided to reduce artifacts. We can also observe from the figure that the DW images are typically very noisy, indicating the great importance of denoising.

In this paper, we propose a group *ℓ*_0_ minimization denoising framework that utilizes tight wavelet frames and takes advantage of the correlation between DW images scanned with neighboring gradient directions. The power of tight wavelet frames lies in their ability to sparsely approximate piecewise smooth functions and the existence of fast decomposition and reconstruction algorithms associated with them. In contrast, TV based methods are effective on restoring images that are piecewise constant, e.g., binary or cartoon-like images. They will, however, cause staircasing effects in images that are not piecewise constant [6].

TV denoising is typically realized by penalizing the *ℓ*_1_-norm of image gradients. Instead of *ℓ*_1_ regularization, which has been shown in the theory of compressed sensing [7] to produce sparse solutions, we opt to use *ℓ*_0_ regularization. In [8], wavelet frame based *ℓ*_0_ regularization shows better edge-preserving quality compared with the conventional *ℓ*_1_ regularization. In [9], iterative hard threshoding algorithms show better performance than iterative soft thresholding algorithms. Based on these facts, we propose a group version of *ℓ*_0_ minimization to take advantage of the correlation between DW images. Extensive experiments were carried out using synthetic data with different levels of noncentral chi (nc-*χ*) noise and real diffusion MRI data. The experimental results demonstrate that the proposed method outperforms TV denoising and non-local means (NLM) denoising [10]. Part of this work has been presented in a workshop [11]. Herein, we provide additional examples, results, derivations, and insights that are not part of the workshop publication. The rest of the paper is organized as follows: In Approach Section, we will provide detailed descriptions for our method. In Experiments Section, we will demonstrate the effectiveness of our method using extensive experiments on synthetic data and real data. In Discussion Section, we will provide in-depth discussions of our method. Finally, we will conclude this work in Conclusion Section.

## Approach

We will provide first a brief introduction to framelets, followed by details on how framelets can be incorporated into an *ℓ*_0_ minimization framework for DWI denoising.

### Tight framelets

A wavelet system $X\subset L_{2}\left(R\right)$ is called a tight wavelet frame of $L_{2}\left(R\right)$ [12] if

$$f=\sum_{g\in X}\left\langle f,g\right\rangle g,\;\forall f\in L_{2}\left(R\right),$$ (1)

where 〈⋅, ⋅〉 is the inner product of $L_{2}\left(R\right)$. It is clear that an orthonormal basis is a tight frame, since the identity hold for arbitrary orthonormal bases in $L_{2}\left(R\right)$. When *X*(Ψ) forms an orthonormal basis of $L_{2}\left(R\right)$, *X*(Ψ) is called an orthonormal wavelet basis. Tight frames are generalization of orthonormal bases with greater redundancy—a property central to sparse representation and often desirable in applications such as denoising [13].

Given a set of generators $\Psi :=\left\{\psi_{1},\ldots ,\psi_{R}\right\}\subset L_{2}\left(R^{d}\right)$, which are desirably (anti)symmetric and compact functions, the corresponding quasi-affine system *X*(Ψ) from level *J* is the collection of dilations and shifts of Ψ:

$$X\left(\Psi \right)=\left\{\psi_{l,r,k}:1\leq r\leq R;l,k\in Z\right\},$$ (2)

with

$$\psi_{l,r,k}≔\{\begin{array}{cc} 2^{\frac{l}{2}}\psi_{r}\left(2^{l}\cdot -k\right), & l\geq J,\\ 2^{\frac{l-J}{2}}\psi_{r}\left(2^{l}\cdot -2^{l-J}k\right), & l<J. \end{array}$$ (3)

When *X*(Ψ) forms a (tight) frame of $L_{2}\left(R\right)$, each function *ψ*_*r*_, *r* = 1, …, *R*, is called a (tight) framelet and the whole system *X*(Ψ) is called a (tight) wavelet frame system. A tight wavelet frame is also called a Parseval frame. Note that in the literature the affine (or wavelet) system, which corresponds to the decimated wavelet (frame) transforms, is commonly used. The quasi-affine system above, introduced and analyzed in [14], corresponds to the undecimated wavelet (frame) transforms and essentially oversamples the wavelet frame system starting from level *J* − 1 and downwards. In this paper, we focus on the quasi-affine system because it has been shown to work better in image restoration [12]. We set *J* = 0 and consider only *l* < 0.

An approach to constructing framelets Ψ is by utilizing multiresolution analysis (MRA) [12]. One starts with a refinable function *ϕ* with refinement mask $a_{0}\in \ell_{2}\left(Z\right)$ satisfying

$$\phi =2\sum_{k\in Z}a_{0}\left[k\right]\phi \left(2\cdot -k\right)$$ (4)

and $\hat{\phi }\left(0\right)=1$, where $\hat{\phi }$ denotes Fourier transform of *ϕ*. The key is to find the masks $a_{r}\in \ell_{2}\left(Z\right)$ that gives

$$\psi_{r}=2\sum_{k\in Z}a_{r}\left[k\right]\phi \left(2\cdot -k\right),\;r=1,2,\cdots ,R.$$ (5)

The finite sequences *a*_1_, …, *a*_*R*_ are called wavelet frame masks, or the high pass filters of the system. The refinement mask *a*_0_ is also known as the low pass filter. The two equations above can be combined by defining *ψ*_0_ ≔ *ϕ*. The unitary extension principle (UEP) [14] provides a general theory for constructing MRA-based tight wavelet frames. That is, as long as {*a*_1_, …, *a*_*R*_} are finitely supported and their Fourier series satisfy

$$\sum_{r=0}^{R}{\left|{\hat{a}}_{r}\left(\xi \right)\right|}^{2}=1\;\text{and}\;\sum_{r=0}^{R}{\hat{a}}_{r}\left(\xi \right)\bar{{\hat{a}}_{r}\left(\xi +\nu \right)}=0,$$ (6)

for all *ν* ∈ {0, *π*} and *ξ* ∈ [−*π*, *π*], the quasi-affine system *X*(Ψ) forms a tight frame in $L_{2}\left(R\right)$.

For example, consider the centered B-splines of order *p*, i.e.,

$$\hat{\phi }\left(\xi \right)=e^{-ij\frac{\xi }{2}}{\left(\frac{\text{sin}(\xi /2)}{\xi /2}\right)}^{p},$$ (7)

with *j* = 0 when *p* is even and *j* = 1 when *p* is odd. The corresponding refinement mask is given as

$${\hat{a}}_{0}\left(\xi \right)=e^{-ij\frac{\xi }{2}}{\text{cos}}^{p}\left(\xi /2\right),$$ (8)

and the *p* wavelet masks as

$${\hat{a}}_{r}\left(\xi \right)≔-i^{r}e^{-ij\frac{\xi }{2}}\sqrt{\left(\begin{array}{c} p\\ r \end{array}\right)}{\text{sin}}^{r}\left(\xi /2\right){\text{cos}}^{p-r}\left(\xi /2\right),$$ (9)

where *r* = 1, 2, …, *p*. It is straightforward to show that the UEP conditions (6) are satisfied. Wavelet frame masks for *p* = 1, 2, 4 are shown in Table 1. It is worth noting that these masks corresponds to differential operators of various orders. For example, for piecewise linear B-spline, the masks *a*_1_ and *a*_2_ correspond to the first order and second order difference operators respectively up to a scaling factor.

**Table 1:** Wavelet frame masks.

Piecewise Constant (*p* = 1)	Piecewise Linear (*p* = 2)	Piecewise Cubic (*p* = 4)
$a_{0}=\frac{1}{2}\left[1,1\right]$	$a_{0}=\frac{1}{4}\left[1,2,1\right]$	$a_{0}=\frac{1}{16}\left[1,4,6,4,1\right]$
$a_{1}=\frac{1}{2}\left[1,-1\right]$	$a_{1}=\frac{\sqrt{2}}{4}\left[1,0,-1\right]$	$a_{1}=\frac{1}{8}\left[-1,-2,0,2,1\right]$
	$a_{2}=\frac{1}{4}\left[-1,2,-1\right]$	$a_{2}=\frac{\sqrt{6}}{16}\left[1,0,-2,0,1\right]$
		$a_{3}=\frac{1}{8}\left[-1,2,0,-2,1\right]$
		$a_{4}=\frac{1}{16}\left[1,-4,6,-4,1\right]$

When a tight wavelet frame is used, the given data is considered to be sampled as a local average

u[k]=⟨f,ϕ(·-k)⟩. (10)

Noting that [12]

$$\left\langle f,\psi_{l-1,r,k}\right\rangle =\sum_{k^{\prime }\in Z}a_{l,r}\left[k^{\prime }\right]\left\langle f,\psi_{l,0,k+k^{\prime }}\right\rangle ,$$ (11)

where the dilated sequence is defined as

$$a_{l,r}\left[k\right]=\{\begin{array}{cc} a_{r}\left[2^{l}k\right], & k\in 2^{-l}Z,\\ 0, & k\notin 2^{-l}Z. \end{array}$$ (12)

The decomposition and reconstruction down to level −*L* [12], i.e.,

$$P_{0}f=P_{-L}f+\sum_{r=1}^{R}\sum_{j=-L}^{-1}\sum_{k\in Z}\left\langle f,\psi_{r,j,k}\right\rangle \psi_{r,j,k},$$ (13)

where

$$P_{l}f=\sum_{r=1}^{R}\sum_{j<l}\sum_{k\in Z}\left\langle f,\psi_{r,j,k}\right\rangle \psi_{r,j,k},$$ (14)

can be realized with convolution using the masks. Denoting by *W* the *L*-level framelet decomposition, i.e.,

$$Wf={\left(\ldots ,W_{l,r}f,\ldots \right)}^{⊤}\;\text{for}\;\left(l,r\right)\in B_{L},$$ (15)

with

$$\begin{array}{c} \begin{array}{c} B_{L}≔\{\left(1,1\right),\left(1,2\right),\cdots ,\left(1,R\right),\\ (2,1),\cdots ,(L,R)\}\cup \{(L,0)\}, \end{array} \end{array}$$ (16)

we have

$$W_{l,r}f=a_{-l,r}*a_{-l+1,0}*\cdots *a_{0,0}*f,$$ (17)

where * denotes the convolution operator. If we use *W*^⊤^ to denote the framelet reconstruction, we have *W*^⊤^*W* = *I*, i.e., *f* = *W*^⊤^*Wf*.

Given a 1-dimensional framelet system for $L_{2}\left(R\right)$, the *d*-dimensional tight wavelet frame system for $L_{2}\left(R^{d}\right)$ can be easily constructed by using tensor products of the 1-dimensional framelets [12].

### Problem formulation

Given a multi-channel or vector-valued image *f* of an arbitrary dimension with voxel *i* ∈ {1, …, N} consisting of vector *f*_*i*_ ∈ ℜ^*M*^, where *N* is the number of voxels and *M* is the number of channels, we are interested in restoring its denoised counterpart *u* by solving the following problem:

$$\underset{u}{\text{min}}\{\Phi \left(u\right)={∥u-f∥}_{2}^{2}+\sum_{i,g,l,r}\lambda_{g,l,r}{∥\sqrt{\sum_{m}w_{g,m}^{2}{∥{\left(W_{l,r}u^{\left(m\right)}\right)}_{i}∥}_{2}^{2}}∥}_{0}\}.$$ (18)

Here, *u*^(*m*)^ is the *m*-th channel of *u*. The regularization term is in fact a summation of *G* terms, each of which grouping a number of channels. The *g*-th grouping (with associated tuning parameter λ_*g*,*l*,*r*_), where *g* = {1, 2, …, *G*}, is defined according to the set of weights {*w*_*g*,*m*_}, where *m* ∈ {1, 2, …, *M*}. Channels with *w*_*g*,*m*_ ≠ 0 are included in the grouping and their weighted framelet coefficients are jointly considered via *ℓ*_2_-norm for penalization. The different groups can possibly overlap, implying that each channel can be included in different groups at the same time. This is in spirit similar to the overlapped group Lasso [15]. We set

$$\begin{array}{c} \lambda_{g,l,r}=\{\begin{array}{cc} \lambda {\left(\sum_{m}w_{g,m}^{2}\right)}^{\frac{1}{2}}, & l,r\neq 0,\\ 0, & \text{otherwise}. \end{array} \end{array}$$ (19)

Here λ is a constant that can be set independently of the weights.

### Optimization

Problem (18) can be solved effectively using penalty decomposition (PD) [16]. Defining auxiliary variables

$${\left(v_{g,m,l,r}\right)}_{i}≔w_{g,m}{\left(W_{l,r}u^{\left(m\right)}\right)}_{i},$$ (20)

this amounts to minimizing the following objective function with respect to *u* and *v* ≔ {*v*_*g*,*m*,*l*,*r*_}:

$$\begin{array}{ll} L_{\mu }\left(u,v\right)= & {∥u-f∥}_{2}^{2}\sum_{i,g,l,r}\lambda_{g,l,r}{∥\sqrt{\sum_{m}{∥{\left(v_{g,m,l,r}\right)}_{i}∥}_{2}^{2}}∥}_{0}\\ & +\frac{\mu }{2}\sum_{i,g,m,l,r}{∥w_{g,m}{\left(W_{l,r}u^{\left(m\right)}\right)}_{i}-{\left(v_{g,m,l,r}\right)}_{i}∥}_{2}^{2}. \end{array}$$ (21)

In PD, we (i) alternate between solving for *u* and *v* using block coordinate descent (BCD). Once this converges, we (ii) increase *μ* > 0 by a multiplicative factor *δ* > 1 and repeat step (i). This is repeated until increasing *μ* does not result in further changes to the solution [16]. See Algorithm 1 for a summary of the algorithm. Convergence analysis is provided in the S1 Appendix.

#### First subproblem

We solve for *v* in the first problem, i.e., min_*v*_ *L*_*μ*_(*u*, *v*). This is a group *ℓ*_0_ problem and the solution can be obtained via hard-thresholding:

$${\left(v_{g,m,l,r}\right)}_{i}=\{\begin{array}{cc} w_{g,m}{\left(W_{l,r}u^{\left(m\right)}\right)}_{i}, & \text{if}\;{\left(h_{g,l,r}\right)}_{i}\geq \frac{2\lambda_{g,l,r}}{\mu },\\ 0, & \text{if}\;\text{otherwise,} \end{array}$$ (22)

where

$${\left(h_{g,l,r}\right)}_{i}=\sum_{m^{\prime }}{∥w_{g,m^{\prime }}{\left(W_{l,r}u^{\left(m^{\prime }\right)}\right)}_{i}∥}_{2}^{2}.$$ (23)

This subproblem can be replaced using soft-thresholding to obtain an *ℓ*_1_ version of the algorithm.

#### Second subproblem

By taking the partial derivative with respect to *u*^(*m*)^, the solution to the second subproblem, i.e., min_*u*_ *L*_*μ*_(*u*, *v*), is for each *m*

$$(I+\frac{\mu }{2}\sum_{g,l,r}w_{g,m}^{2}W_{l,r}^{⊤}W_{l,r})u^{\left(m\right)}=f^{\left(m\right)}+\frac{\mu }{2}\sum_{g,l,r}w_{g,m}\;W_{l,r}^{⊤}v_{g,m,l,r},$$ (24)

where we have dropped the subscript *i* for notation simplicity. Note that since we have $\sum_{l,r}W_{l,r}^{⊤}W_{l,r}=I$, the the problem can be simplified to

$$(1+\frac{\mu }{2}\sum_{g}w_{g,m}^{2})u^{\left(m\right)}=f^{\left(m\right)}+\frac{\mu }{2}\sum_{g,l,r}w_{g,m}\;W_{l,r}^{⊤}v_{g,m,l,r}.$$ (25)

Solving the above equation for *u*^(*m*)^ is trivial and involves only simple division.

**Algorithm 1**: Penalty Decomposition (PD)

**Data**     : Multi-channel image *f*.

**Parameters**  : Tuning parameter λ; initial penalty factor *μ*_0_ > 0; multiplicative factor *δ* > 1; BCD tolerance *ϵ*_BCD_; PD tolerance *ϵ*_PD_.

**Initialization**  : Iteration index *k* = 0; initial solution *u*^0,0^; a constant ϒ ≥ Φ(*u*^0,0^).

**Output**    : Denoised image *u*.

/* Main Steps */

(1) For a fixed *μ*_*k*_, obtain BCD solution (*u*^*k*^, *v*^*k*^) for ${\text{min}}_{u,v}\;L_{\mu_{k}}\left(u,v\right)$. That is, set *k*′ = 0 and iterate the following steps:

 (1a) Solve $v^{k,k^{\prime }+1}\in {\text{arg}\;\text{min}}_{v}\;L_{\mu_{k}}\left(u^{k,k^{\prime }},v\right)$.

 (1b) Solve $u^{k,k^{\prime }+1}={\text{arg}\;\text{min}}_{u}\;L_{\mu_{k}}\left(u,v^{k,k^{\prime }+1}\right)$.

 (1c) If *u*^*k*,*k*′+1^ satisfies the BCD stopping criterion

$$\frac{∥u^{k,k^{\prime }}-u^{k,k^{\prime }+1}{∥}_{2}}{\text{max}(∥u^{k,k^{\prime }}{∥}_{2},1)}\;\leq \;\epsilon_{\text{BCD}},$$

  set (*u*^*k*^, *v*^*k*^) = (*u*^*k*,*k*′+1^, *v*^*k*,*k*′+1^) and go to Step (2).

 (1d) Set *k*′ ← *k*′ + 1 and go to Step (1).

(2) If *u*^*k*^ satisfies the PD stopping criterion

$$\frac{∥u^{k}-u^{k+1}{∥}_{2}}{\text{max}(∥u^{k}{∥}_{2},1)}\;\leq \;\epsilon_{\text{PD}},$$

  stop and output *u*^*k*^. Otherwise, set *μ*_*k*+1_ = *δμ*_*k*_.

(3) If ${\text{min}}_{v}\;L_{\mu_{k+1}}\left(u^{k},v\right)>ϒ$, set *u*^*k*+1,0^ = *u*^0,0^. Otherwise, set *u*^*k*+1,0^ = *u*^*k*^.

(4) Set *k* → *k* + 1 and go to Step (1).

### Setting the weights

In our case, each channel corresponds to a DW image. In setting the weights {*w*_*g*,*m*_}, we note that the weights should decay with the dissimilarity between gradient directions associated with a pair of DW images. To reflect this, we let *G* = *M* and set, for *g*, *m* ∈ {1, …, *M*},

$$w_{g,m}=\{\begin{array}{cc} e^{\kappa [{\left(\nu_{m}^{⊤}\nu_{g}\right)}^{2}-1]}, & |\nu_{m}^{⊤}\nu_{g}|⩾\text{cos}\left(\theta \right),\\ 0, & \text{otherwise}, \end{array}$$ (26)

where *κ* ≥ 0 is a parameter that determines the rate of decay of the weight. The exponential function is in fact modified from the probability density function of the Watson distribution [17] with concentration parameter *κ*. Essentially, this implies that for the *g*-th DW image acquired at gradient direction *ν*_*g*_, there is a corresponding regularization group that includes a set of images with associated weights {*w*_*g*,*m*_}. The weight is maximal at *w*_*g*,*g*_ = 1 and is attenuated when *m* ≠ *g*. Weights of images scanned at a gradient direction with angle greater than *θ* in relation to *ν*_*g*_ are set to 0, and the respective images are hence discarded from the group. We set *θ* = 30°.

### Debiasing

The magnitude of the complex MR signal is commonly used because the phase of the complex signal is highly sensitive to many experimental factors [18, 19]. The magnitude MR signal is not affected by the phase error and it follows a nc-*χ* distribution [20, 21] rather than a Gaussian distribution and bias correction needs to be carried out especially when the SNR is low [18]. Bias correction can be performed before [22] or after [23] denoising. In our case, we adopted the latter for unbiased noise reduction [23].

## Experiments

The main goal in the following experiments is to demonstrate that denoising performance can be improved by using

1. UEP-based tight wavelet frames, which avoids the staircasing effect;
2. *ℓ*_0_ over *ℓ*_1_ regularization;
3. Collaborative utilization of angularly neighboring DW images.

Unless stated otherwise, we used the piecewise linear tight wavelet frame with *L* = 2 levels of decomposition. The optimal λ values for *ℓ*_0_ and *ℓ*_1_ were in (1, 8], determined using grid search from 0.2 to 50 in steps of 0.2 based on the maximal peak signal-to-noise ratio (PSNR) defined as

$$\text{PSNR}=10\cdot {\text{log}}_{10}(\frac{{\text{MAX}}^{2}}{\text{MSE}}),$$ (27)

where MAX is the maximal signal value and MSE is the mean square error.

For debiasing, the noise level is estimated from the image background using the method described in [24]. More advanced noise estimation methods [23, 25] can be used for improved accuracy.

We utilized NLM filtering as a comparison baseline. Following the work presented in [26], we set the patch radius to 1 and search radius to 2.

### Datasets

#### Spiral data

A synthetic dataset of a spiral was generated for quantitative evaluation. The parameters used for synthetic data simulation were consistent with the real data described next: *b* = 2000s/mm^3^, 48 gradient directions, 64 × 64 × 16 voxels with resolution 2 × 2 × 2 mm^3^. Three levels of 32-channel nc-*χ* noise [27] was added: *σ* = 5, 7.5, and 10, corresponding to SNR = 30, 20, 10. SNR is defined as *η*/*σ* [28], where *η* is the true signal value, which in our case is the white matter non-DW signal. The varying curvature reflects the various degree of bending of white matter pathways and gives us a good basis for evaluating how denoising performance changes in different conditions.

#### ISBI phantom

Evaluation was also performed using the realistic diffusion MRI phantom adopted in the ISBI 2013 HARDI challenge (http://hardi.epfl.ch/static/events/2013_ISBI/). A python package, called phantom*α*s [29], was used to generate the noise free phantom, with gradient directions and diffusion weighting consistent with the spiral data described above. Three levels of 32-channel nc-*χ* noise, similar to the spiral data, was added to the noise free phantom.

#### Real data

DW images were acquired using Siemens 3T TRIO MR scanner with the same gradient directions and *b*-value as the spiral data. The imaging protocol is as follows: 128 × 96 imaging matrix, voxel size of 2 × 2 × 2 mm^3^, TE = 97 ms, TR = 11, 300 ms, 32-channel receiver coil. Imaging acquisition was repeatedly performed on the same subject for 8 times. We averaged the 8 sets of DW images and removed the nc-*χ* noise bias to obtain the ground truth for evaluation. Informed written consent was obtained from the subject and the experimental protocol was approved by the Institutional Review Board of the University of North Carolina (UNC) School of Medicine. The study was carried out in accordance with the approved guidelines.

### Results

#### The staircasing effect

**Fig 2:**
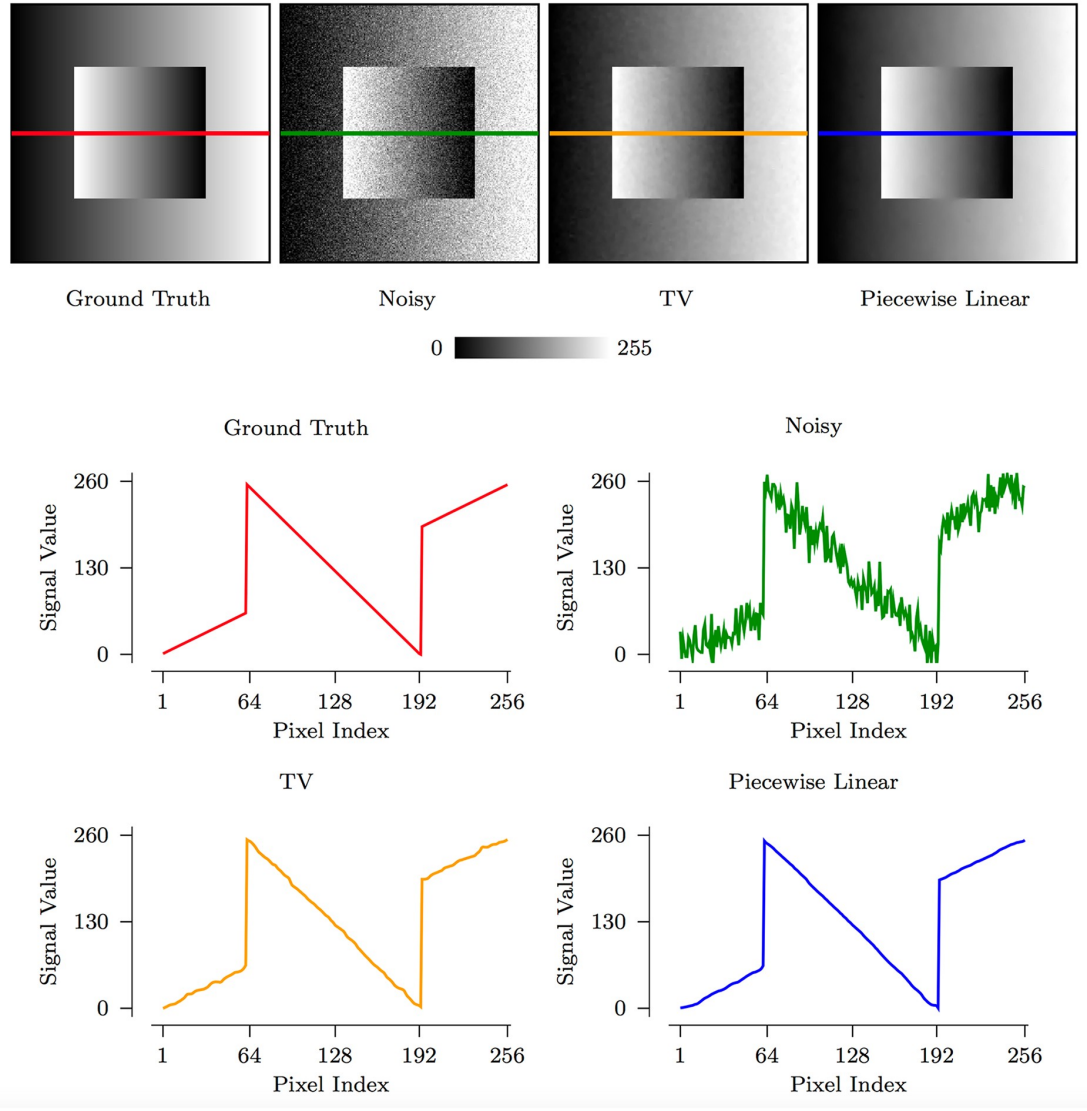
An illustration of how piecewise linear framelet denoising avoids the staircasing artifacts created by TV denoising.

The staircasing effect is often observed in denoising based on TV regularization [30]. The power of tight wavelet frames lies in their ability to sparsely approximate piecewise smooth functions. They are hence better suited for images with gradual intensity changes. In Fig 2, we show an example of how piecewise linear framelet denoising avoids the staircasing effect and results in a smoother image without blocking artifacts. In contrast, TV denoising causes patch artifacts when the image is not piecewise constant.

#### Bias correction

**Fig 3:**
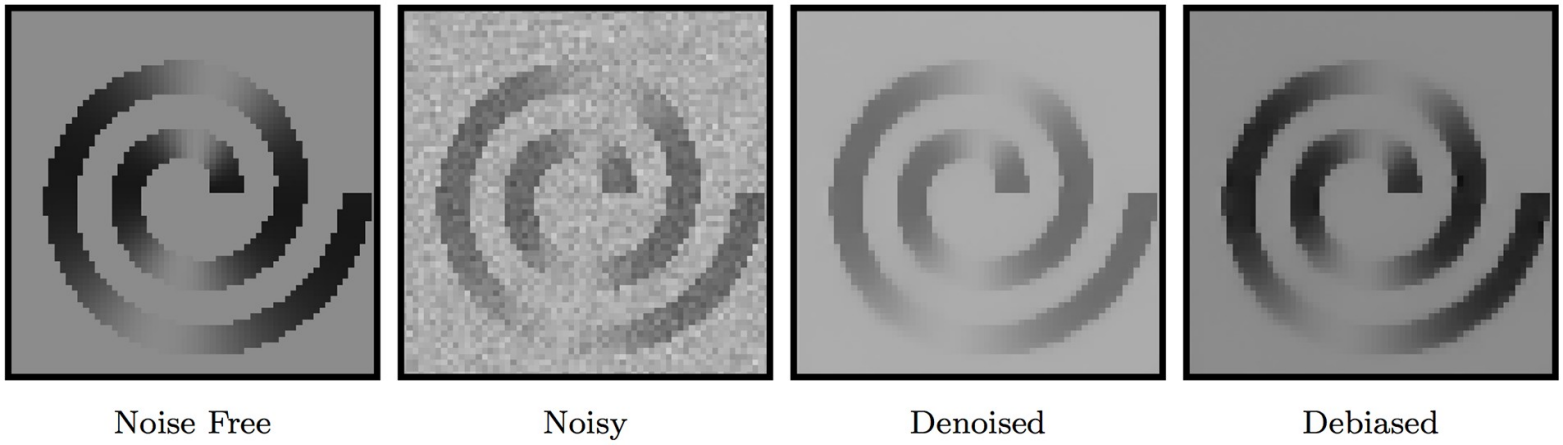
Debiasing the denoising outcome overcomes the noise floor and results in (*σ* = 7.5).

The noise-induced bias on the estimated magnitude signal is especially prominent when the diffusion weighting is high. We removed the nc-*χ* bias using the method described in [27]. Fig 3 indicates that the nc-*χ* noise results in a noise floor especially when the signal is low [27]. This manifests as elevation of intensity value after denoising. Removing the noise bias produces an image that is closer to the ground truth.

#### Type of framelets and number of levels

**Fig 4:**
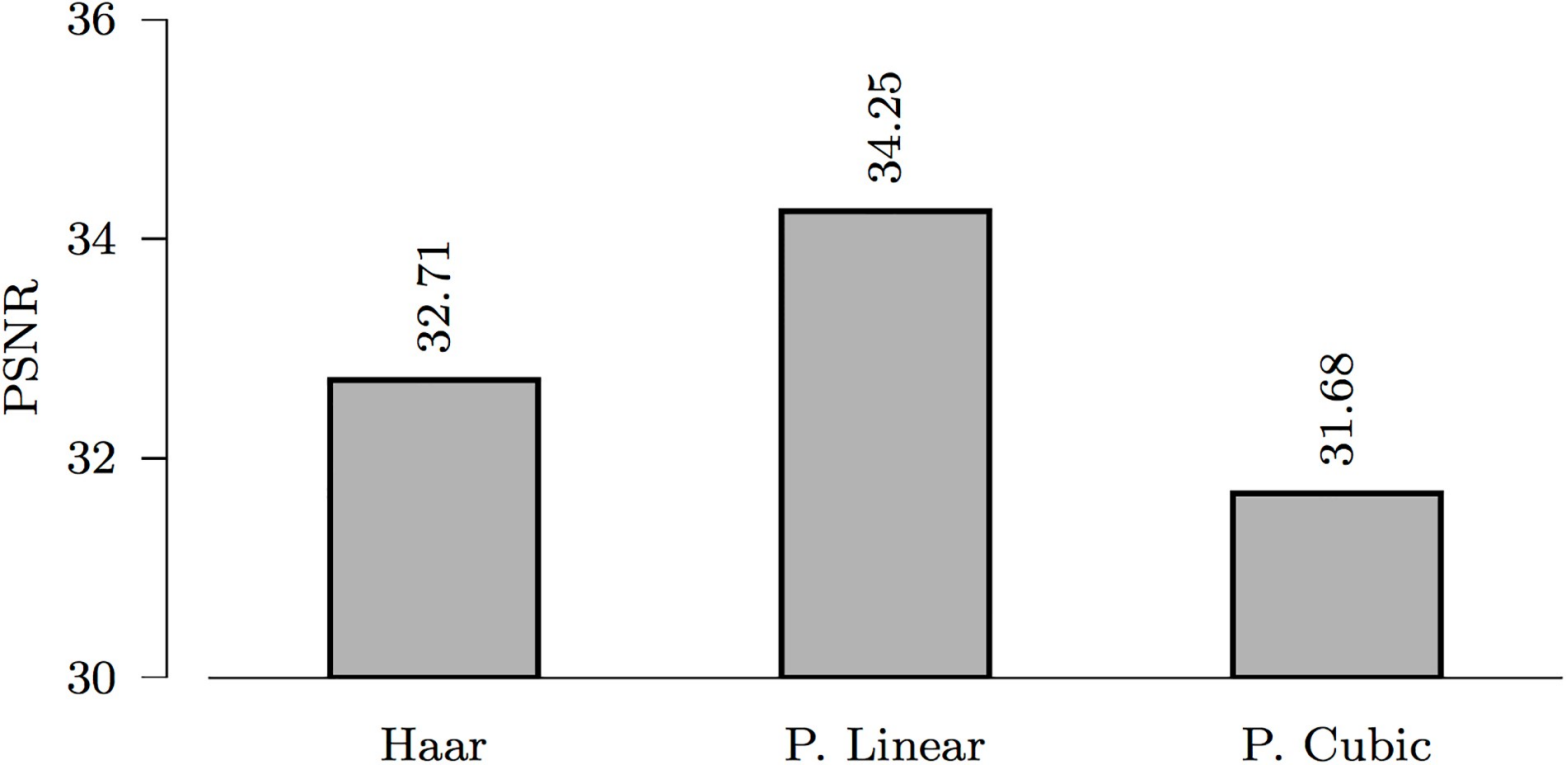
*ℓ*_0_ denoising using Haar, piecewise linear, and piecewise cubic framelets (*L* = 2, *σ* = 5).

Using the spiral data for evaluation, our results shown in Fig 4 indicate that piecewise linear framelet denoising performs better than other types of framelets.

**Fig 5:**
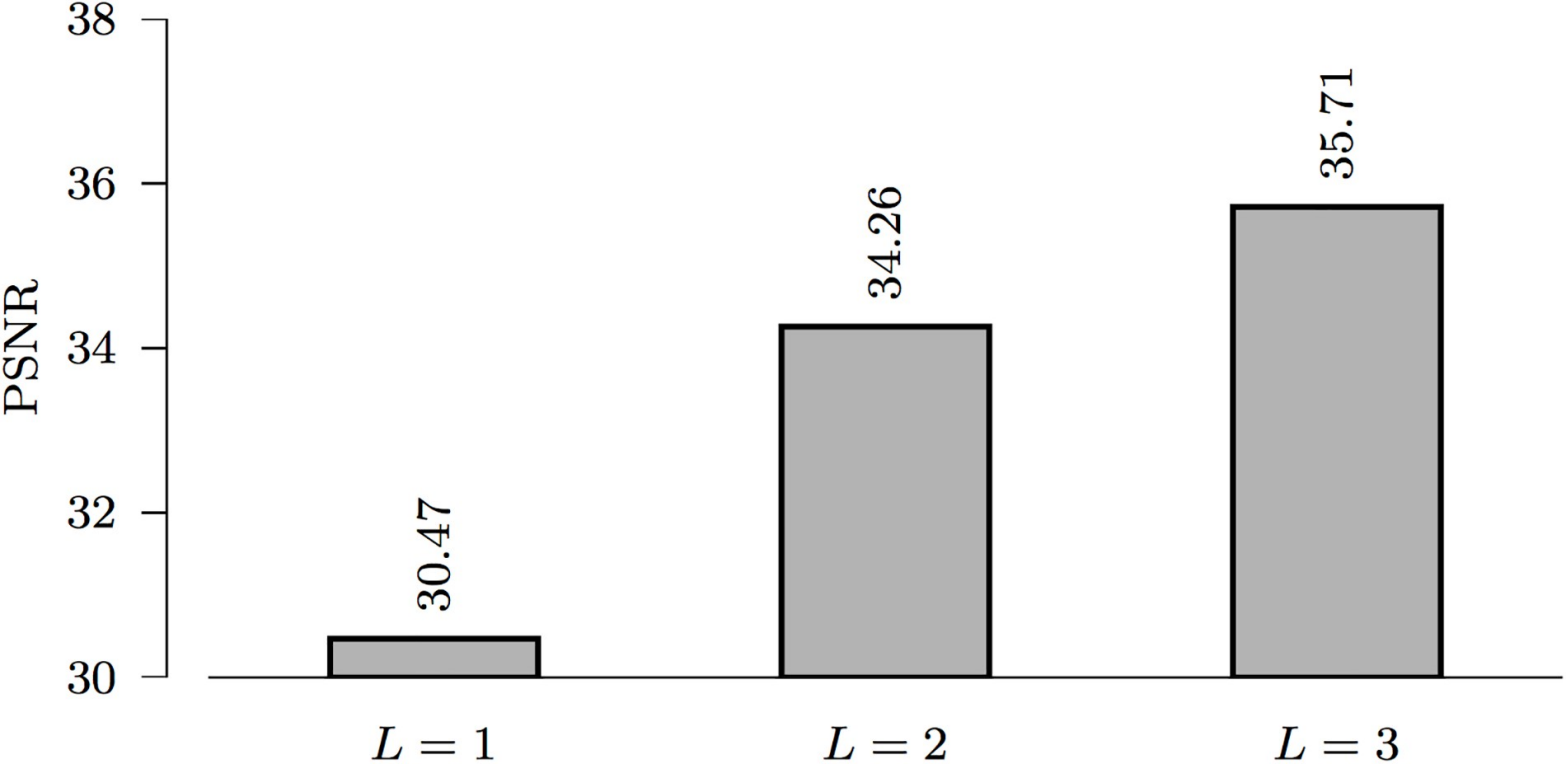
*ℓ*_0_ denoising in relation to the level of decomposition, *L* (piecewise linear framelets, *σ* = 5).

Fig 5 indicates that denoising performance improves with the increase in the number of levels, *L*. However, the time cost increases dramatically with *L*, i.e., 27 s for *L* = 1, 29 s for *L* = 2, and 378 s for *L* = 3 (based on a 4-core Intel i7 processor). Therefore, we choose *L* = 2 for reasonable denoising performance with a reasonable time cost.

#### Effects of grouping

**Fig 6:**
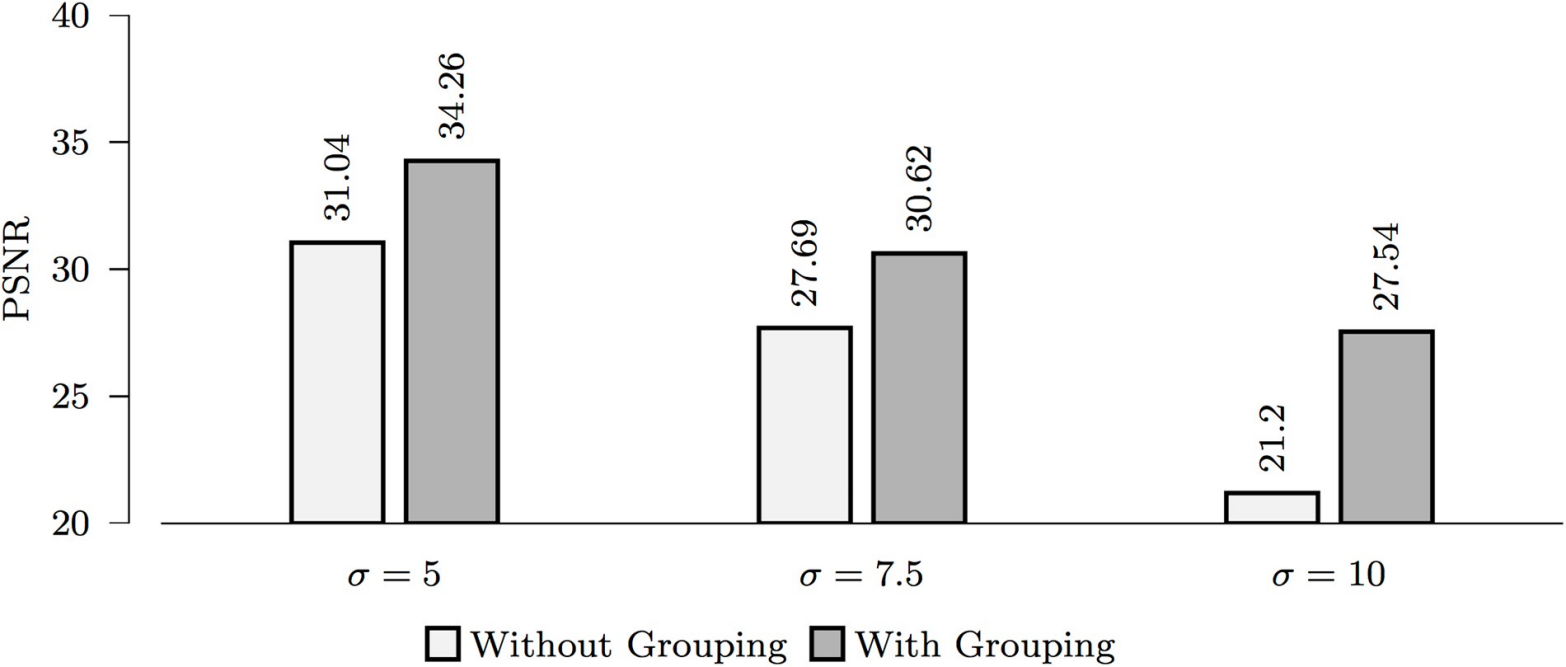
The effects of grouping on denoising.

Fig 6 shows the results of denoising with and without grouping of angularly neighboring images. Grouping can be observed to significantly improve PSNR.

#### Comparison between methods

**Fig 7:**
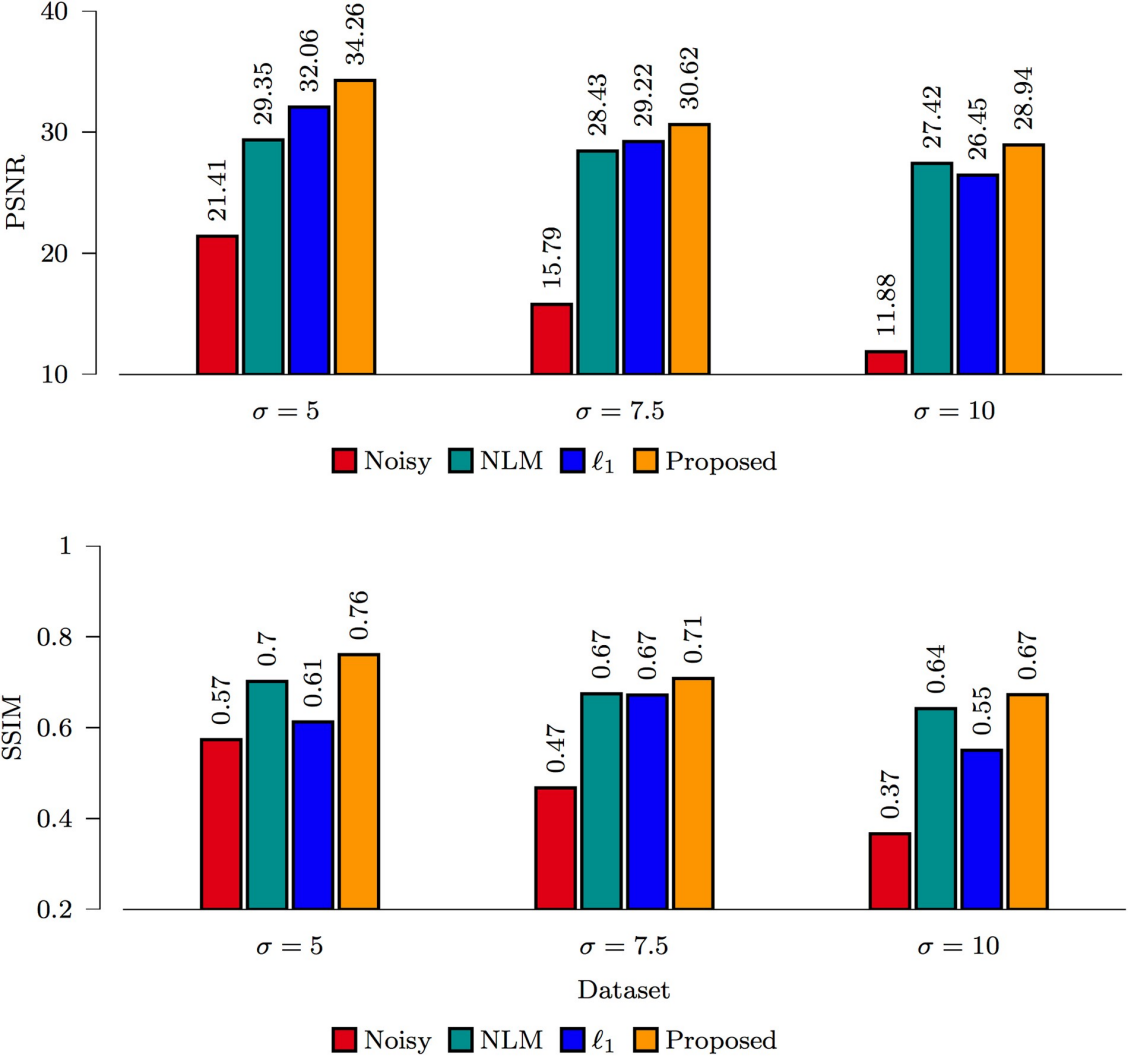
Performance comparison based on the spiral data.

**Fig 8:**
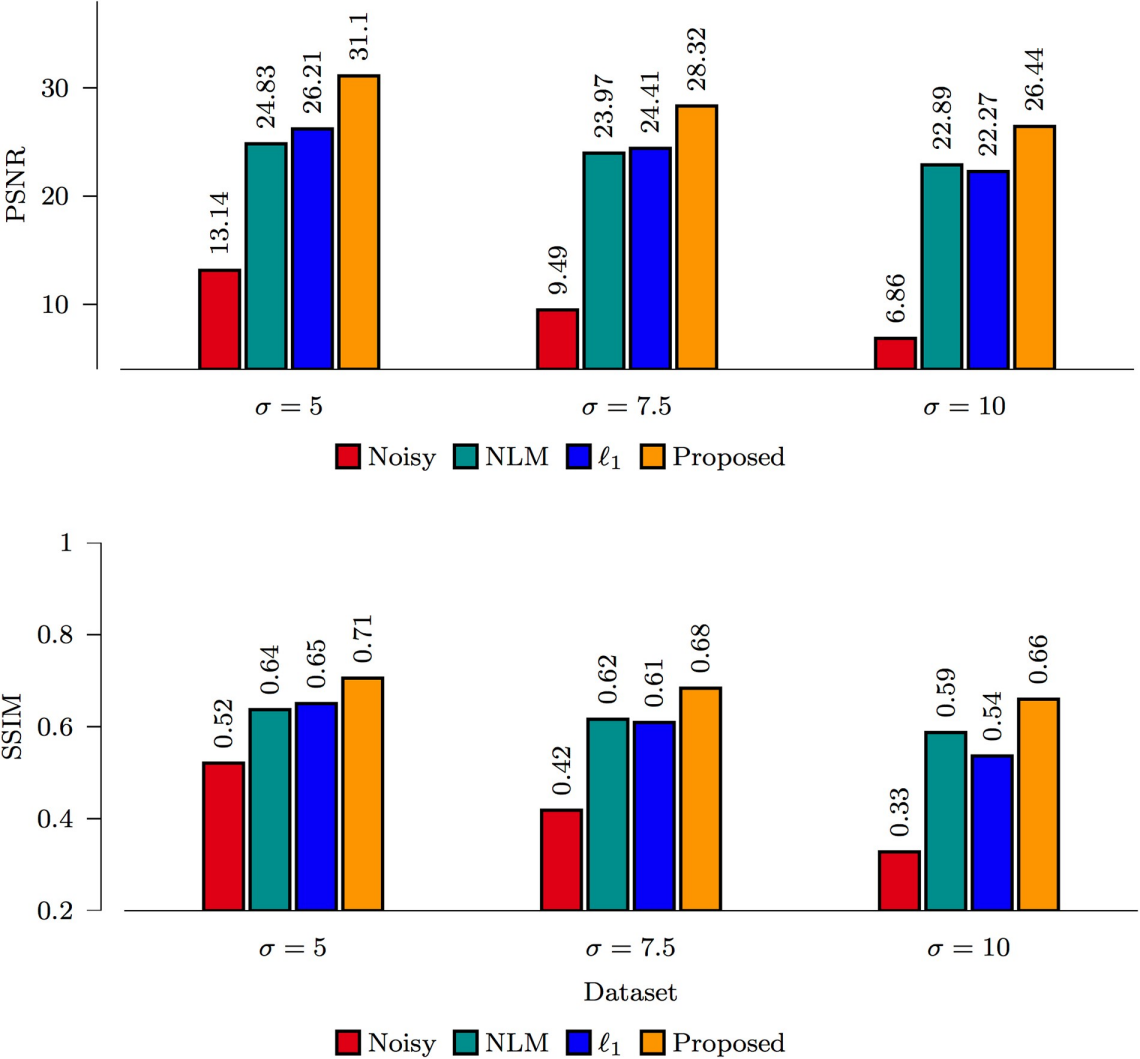
Performance comparison based on the ISBI phantom.

**Fig 9:**
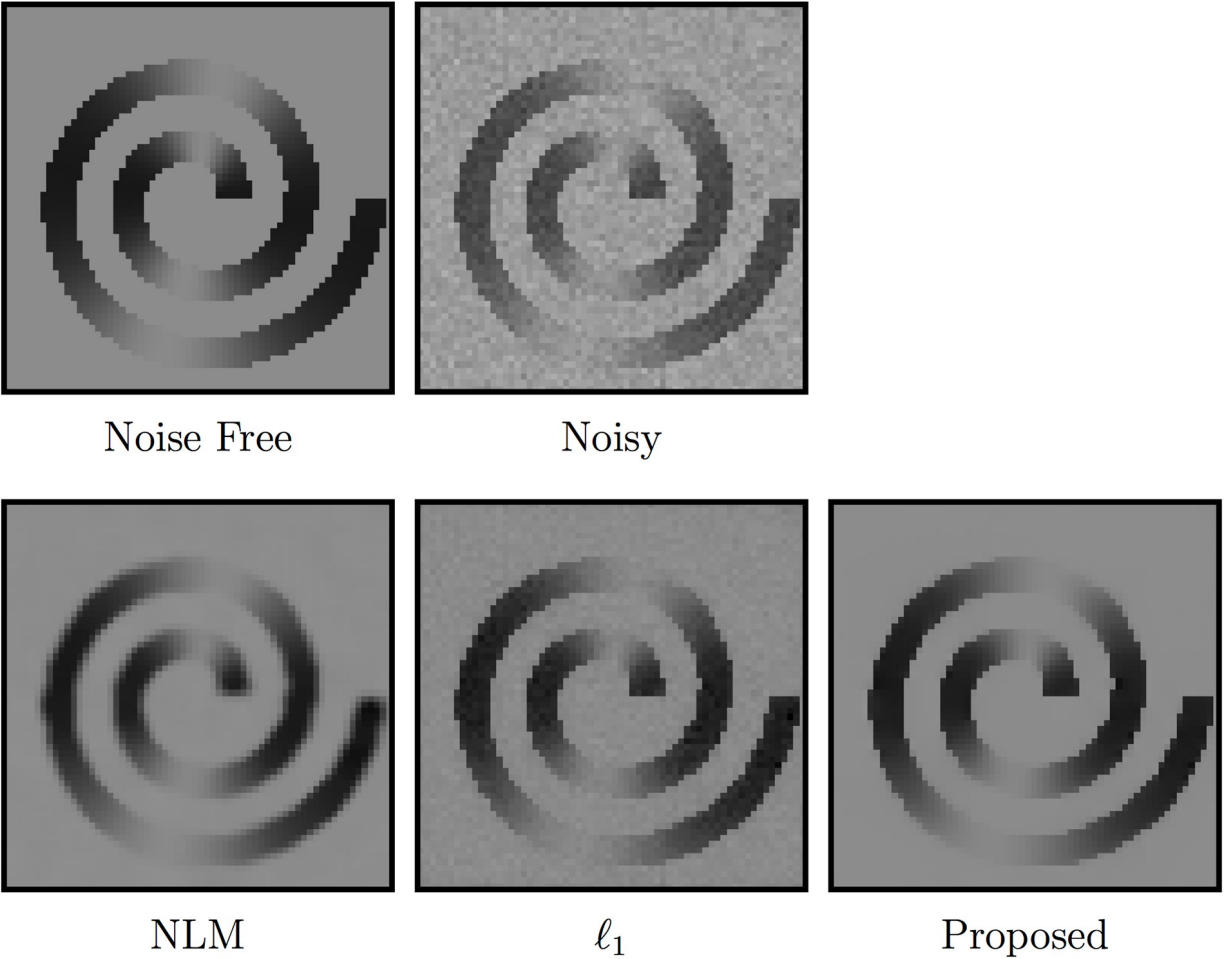
Comparison of denoised DW images given by different methods (*σ* = 5).

**Fig 10:**
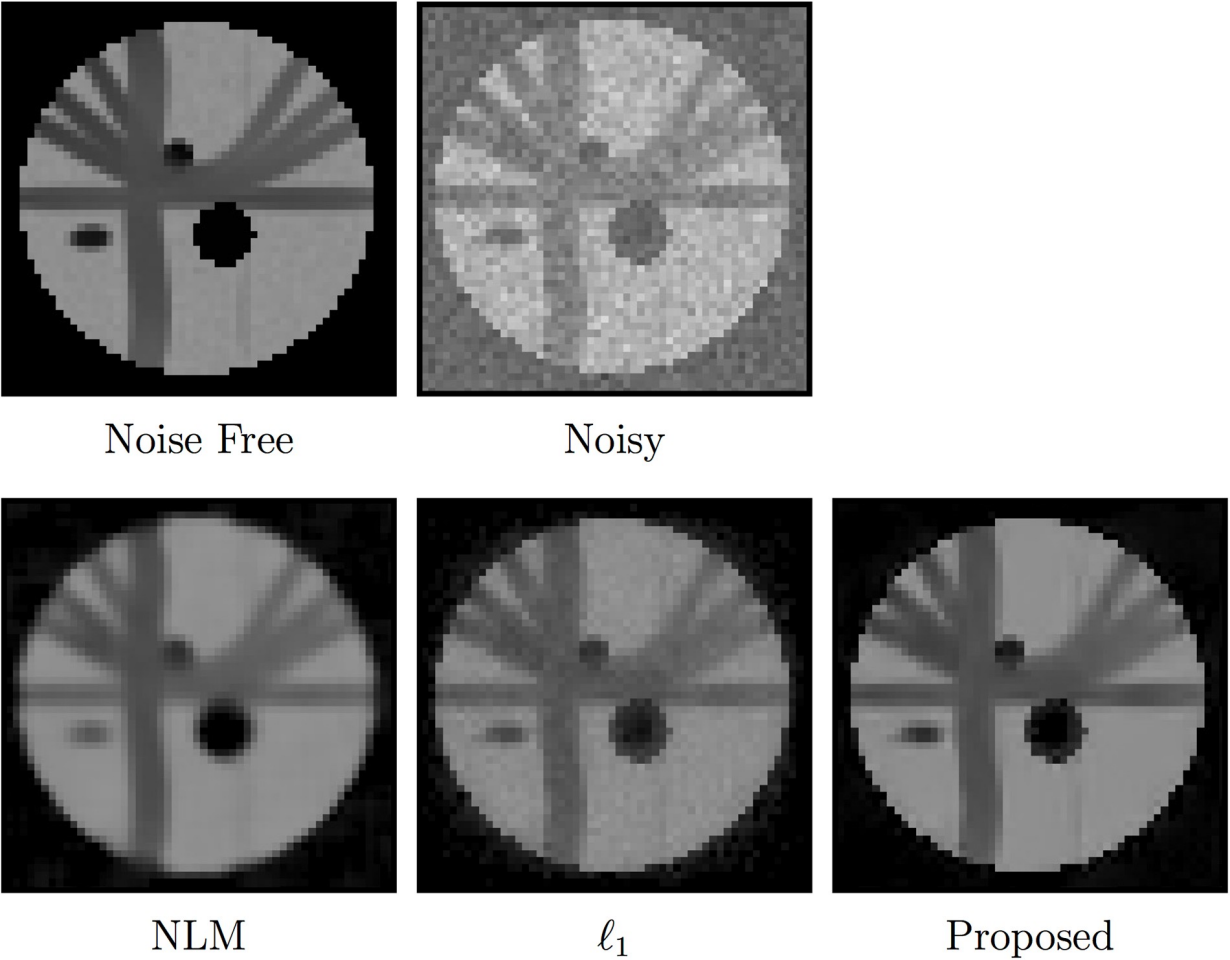
Comparison of denoised DW images given by different methods (*σ* = 7.5).

The PSNR and SSIM [31] results for the spiral data and ISBI phantom, shown in Figs 7 and 8, indicate that the proposed *ℓ*_0_-based framelet method gives the best performance for all noise levels. The DW images, shown in Figs 9 and 10, indicate that both *ℓ*_1_ and *ℓ*_0_ give sharper edges compared with NLM. Noise, however, is not totally removed for the case of *ℓ*_1_. Only *ℓ*_0_ is able to effectively remove noise and preserve edges. Note that, for both synthetic and real data, nc-*χ* bias was removed using the method described in [27].

We also compared the computational times of different methods using the spiral data with a computer equipped with a 4-core Intel i7 processor. The results, shown in Table 2, indicate that our method and *ℓ*_1_ perform more efficiently than NLM.

**Table 2:** Computation times.

	NLM	*ℓ*_1_	Proposed
Time (sec)	186	34	27

#### Real data

**Fig 11:**
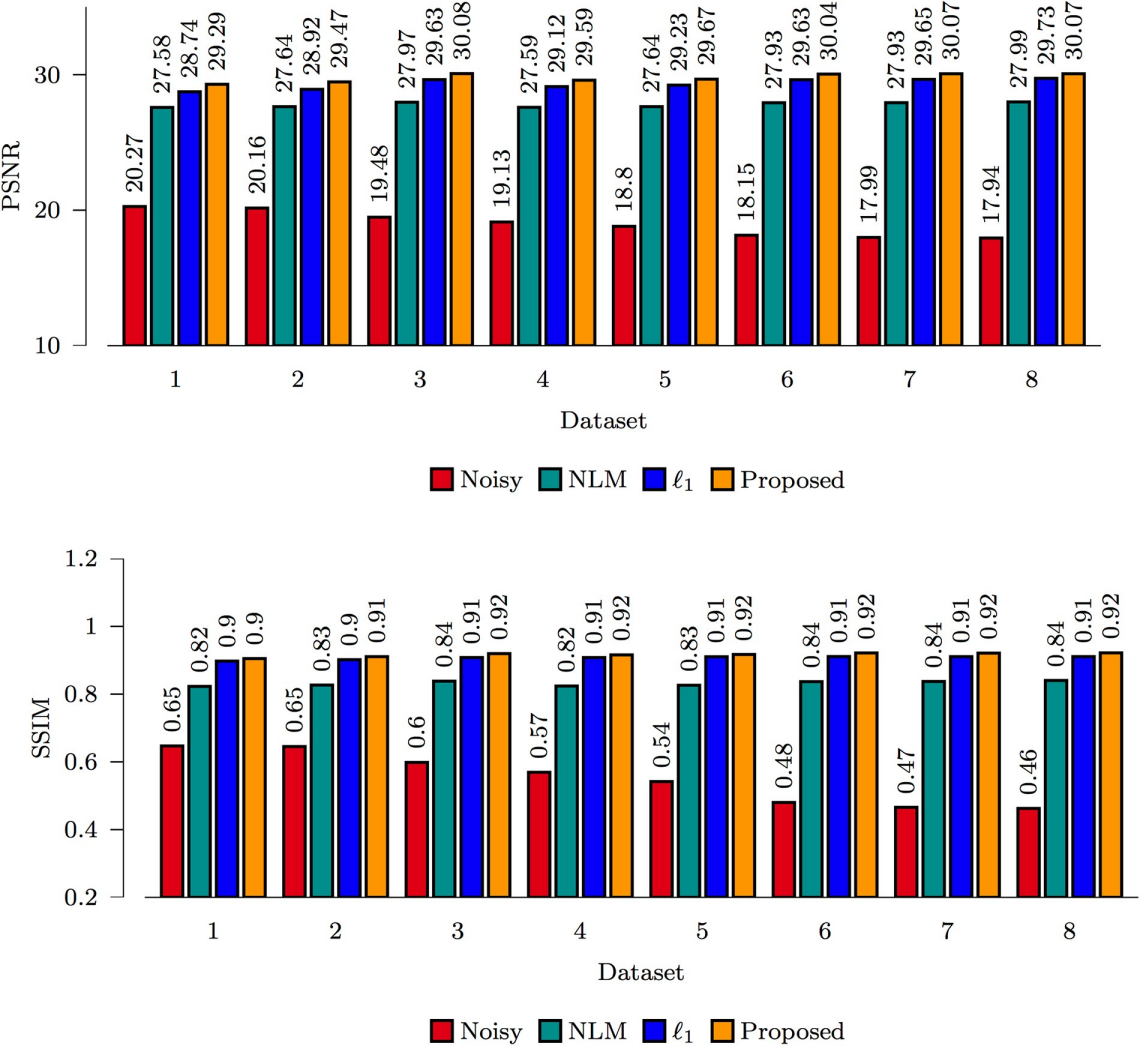
Performance comparison based on the real data.

**Fig 12:**
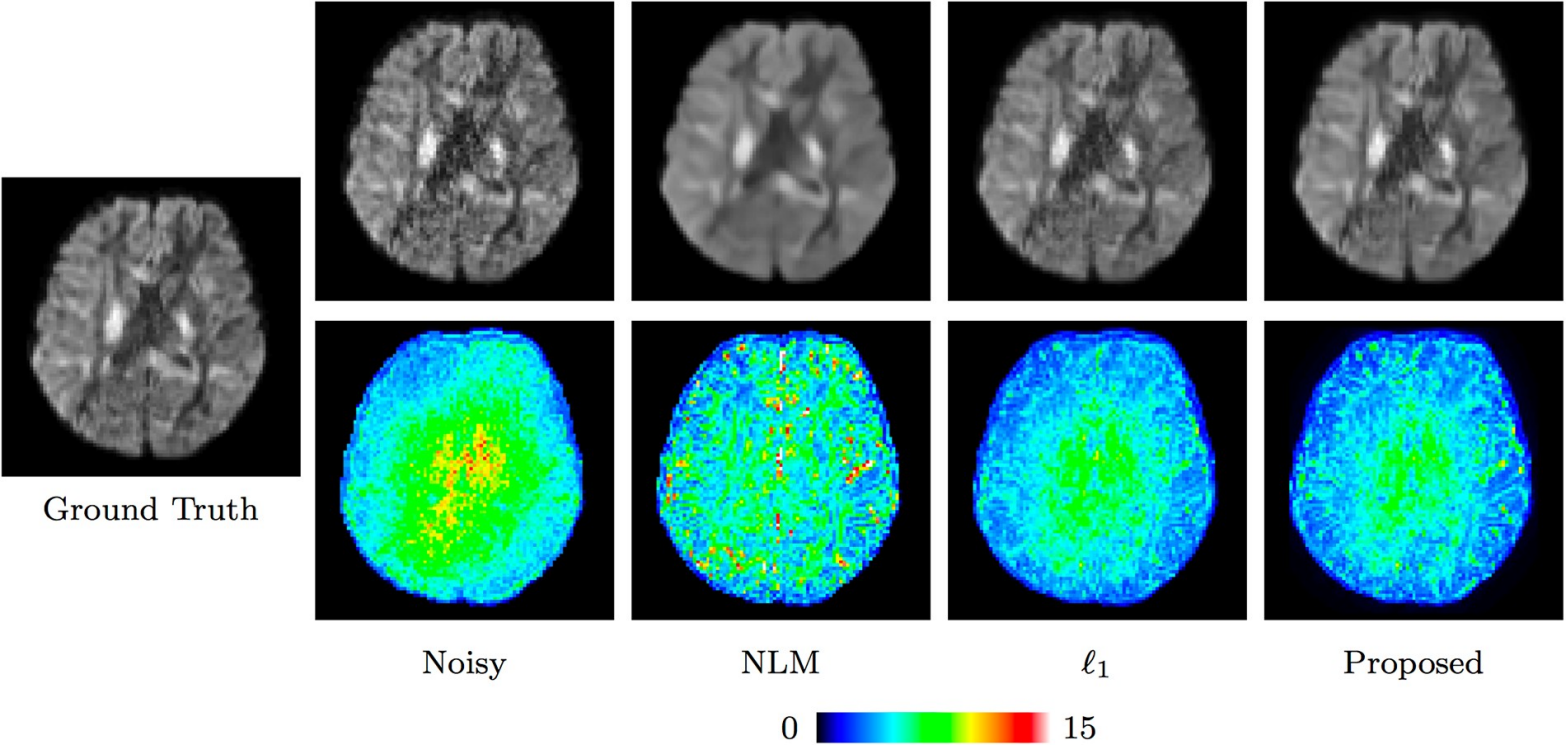
Comparison of denoised DW images using the real data.

For the real data, we used the average image as the ground truth for quantitative evaluations. The results for all 8 datasets, shown in Fig 11, are consistent with Figs 7 and 8, indicating that *ℓ*_0_ gives the best performance. The visual results in Fig 12 indicate that the results given by *ℓ*_0_ is closest to the ground truth. This is confirmed by the root-mean-square error (RMSE) map computed between the denoised data and the ground truth data. In contrast, NLM over-smooths the image and edge information is hence lost.

## Discussion

In this paper, we have introduced a method that harnesses correlations between DW images scanned with similar gradient directions for effective edge-preserving denoising. Our main contributions lie in three aspects. Firstly, UEP was employed to generate frames that were discrete analogues to differential operators of various orders; Secondly, instead of the conventional *ℓ*_1_ regularization, a very efficient method was proposed in order to solve an *ℓ*_0_ denoising problem that involves only thresholding and a trivial inverse problem; Thirdly, DW images acquired using neighboring gradient directions were used for collaborative denoising.

NLM was used as a comparison baseline in our evaluation. However, similar to [22, 32], we found the performance of NLM to be unsatisfactory. NLM can be improved by designing better metrics for patch matching, instead of the conventional Euclidean distance. For instance, inspired by the human visual system, Foi and Boracchi [33] proposed a patch foveation operator for measuring patch distance. Baselice [34] proposed to measure pixel using the Kolmogorov—Smirnov distance, showing promising performance in reducing speckle noise in ultrasound images. NLM can be further improved by extending its search volume. For instance, collaborative NLM [35] extended the search volume to a number of co-denoising images to enrich the similar information used in noise reduction. Chen et al. [36, 37] proposed to improve NLM by considering the similar information in both spatial domain and diffusion wavevector domain. This idea was further employed to improve atlas building [38] and resolution enhancement [39].

## Conclusion

In conclusion, we have proposed a method to remove the noise in DW images. The proposed method takes advantage of multi-channel framelet and the correlations among DW images for effective noise removal. The associated *ℓ*_0_ optimization problem is solved by an effective iterative hard thresholding algorithm. Extensive experiments on synthetic data and real data demonstrate the advantage of our method over various noise reduction methods, including TV regularization, NLM, and the *ℓ*_1_ counterpart of our method.

## Supporting information

S1 Appendix Proof of convergence. (PDF)
