# Supplementary material for: Multi-channel framelet denoising of diffusion-weighted images
Source: PLoS One. 2019 Feb 6;14(2):e0211621. doi: 10.1371/journal.pone.0211621 (PMC6364918; doi:10.1371/journal.pone.0211621)
Supplement: S1 Appendix — (PDF) [file pone.0211621.s001.pdf]

Supporting Information for "Multi-Channel Framelet Denoising of Diffusion-Weighted Images"

Geng Chen, Jian Zhang, Yong Zhang, Bin Dong, Dinggang Shen, Pew-Thian Yap

S1 Appendix. Proof of Convergence.

For conciseness, we represent the multi-channel image as a matrix $F = [f^{(1)}\ f^{(2)} \dots f^{(M)}] \in \Re^{N \times M}$, then convert problem (18) in the main text to the following form:

$$\begin{array}{rl} \min_U & \|U - F\|_F^2 + \sum_g \|V_g\|_{2,0,\Lambda_g} \\ \text{s.t.} & V_g = WUD_g,\ \text{for } g = 1, \dots, G \end{array} \tag{1}$$

where Λ_g is a vector containing the λ's associated with the g-th group. D_g is a diagonal matrix that contains the group weights. $\| \cdot \|_{2,0,\Lambda_g}$ is $l_{2,1}$-norm weighted by Λ_g:

$$\|V_g\|_{2,0,\Lambda_g} = \sum_i \Lambda_{g,i} \| \|V_{g,i}\|_2 \|_0. \tag{2}$$

W is the framelet decomposition operator with $W^\top W = I$. To apply the penalty decomposition method, we first write the following quadratic penalty function for (1)

$$L_\mu(U, V) = \|U - F\|_2^2 + \frac{\mu}{2} \sum_g \|WUD_g - V_g\|_F^2 + \sum_g \|V_g\|_{2,0,\Lambda_g}, \tag{3}$$

where $V = \{V_1, V_2, \dots, V_g, \dots, V_G\}$. To solve (3), the BCD method alternatively solves the two problems below:

$$\begin{aligned} V_g^{k+1} &\in \operatorname{Arg} \min_{V_g} \frac{\mu}{2} \|WU^k D_g - V_g\|_F^2 + \|V_g\|_{2,0,\Lambda_g}, \\ U^{k+1} &= \arg \min_U \|U - F\|_F^2 + \frac{\mu}{2} \sum_g \|WUD_g - V_g^{k+1}\|_F^2. \end{aligned} \tag{4}$$

For the first problem of (4), we have a closed form solution given by

$$V_g^{k+1} \in H_{\tilde{\Lambda}_g}(WU^k D_g) \quad \text{with} \quad \tilde{\Lambda}_{g,i} := \sqrt{\frac{2\Lambda_{g,i}}{\mu}} \tag{5}$$

where $H_\gamma(\cdot)$ denotes a row-wise hard thresholding operator with threshold γ:

$$[H_\gamma(Z)]_i = \begin{cases} 0 & \text{if } \|Z_i\|_2 < \gamma_i, \\ Z_i & \text{if } \|Z_i\|_2 \geq \gamma_i. \end{cases} \tag{6}$$

The second problem of (4) is convex, unconstrained and quadratic. We first derive the convergence results for the inner loop of the BCD method [1].

Theorem 1. *Assume the sequence $\{(U^q, V^q)\}$ generated by the BCD method described in (4) is bounded and then any accumulation point of the sequence $\{(U^q, V^q)\}$ is a local minimizer of $p_\mu(U, V)$.*

Proof. Suppose that (U^*, V^*) is a limit point of the sequence $\{(U^q, V^q)\}$. Therefore, there exists a subsequence $\{(U^{q'}, V^{q'})\}$ converging to (U^*, V^*). Using (5) and the definition of the hard thresholding operator, we can observe that

$$V_g^* = \lim_{q' \to \infty} V_g^{q'+1} \in H_{\tilde{\Lambda}}(\lim_{q' \to \infty} WU^{q'+1} D_g) = H_{\tilde{\Lambda}}(WU^* D_g). \tag{7}$$

In view of $W^T W = I$ and the optimality condition of the second subproblem of (4), one can see that

$$U^{q'} \left[I + \frac{\mu}{2} \sum_g D_g D_g^\top \right] = F + \frac{\mu}{2} \sum_g W^T V_g^{q'} D_g^\top. \tag{8}$$

For the sake of conciseness, we let

$$\begin{aligned} C &:= \left[I + \frac{\mu}{2} \sum_g D_g D_g^\top \right]^{-1}, \\ X &:= FC, \\ Y^{q'} &:= \frac{\mu}{2} \left(\sum_g W^\top V_g^{q'} D_g^\top C \right). \end{aligned}$$

In addition, let Y^* be the cluster point of $Y^{q'}$ and then it follows from (8) that

$$U^* = X + Y^*. \tag{9}$$

In view of the above two relations, i.e., (7) and (9), one can immediately conclude that $\{(U^*, V^*)\}$ is a fixed point of (4).

Next we show that $\{(U^*, V^*)\}$ is a local minimizer of $p_\mu(U, V)$. By applying (5), the first subproblem of (4) leads to

$$V_g^* \in H_{\tilde{\Lambda}_g} \left(WU^* D_g \right). \tag{10}$$

The second subproblem of (4) gives us

$$2(U^* - F) + \mu \sum_g W^\top (WU^* D_g - V_g^*) D_g^\top = 0. \tag{11}$$

Define index sets

$$\begin{aligned} \Gamma_0 &:= \{(g, i) : \|V_{g,i}^*\|_2 = 0\}, \\ \Gamma_1 &:= \{(g, i) : \|V_{g,i}^*\|_2 \neq 0\}. \end{aligned}$$

It then follows from (6) and (10) that

$$V_{g,i}^* = \begin{cases} 0 & \text{if } i \in \Gamma_0, \\ (WU^* D_g)_i & \text{if } i \in \Gamma_1. \end{cases} \tag{12}$$

Defining $\langle A, B \rangle = \mathrm{Tr}(AB^\top)$, $\|A\|_F^2 = \langle A, A \rangle$, and considering a small variation

$(\partial U, \partial V)$ and we have

$$\begin{aligned}
&L_\mu(U^* + \partial U, V^* + \partial V) \\
&\quad = \|U^* + \partial U - F\|_F^2 \\
&\qquad + \frac{\mu}{2} \sum_g \|V_g^* + \partial V_g - W(U^* + \partial U) D_g\|_F^2 \\
&\qquad + \sum_g \|V_g^* + \partial V_g\|_{2,0,\Lambda_g} \\
&\quad = \|U^* - F\|_F^2 + 2\langle \partial U, U^* - F\rangle + \|\partial U\|_F^2 \\
&\qquad + \frac{\mu}{2} \sum_g \|V_g^* - WU^* D_g\|_F^2 \\
&\qquad + \mu \sum_g \langle V_g^* - WU^* D_g, \partial V_g - W\partial U D_g\rangle \\
&\qquad + \frac{\mu}{2} \sum_g \|\partial V_g - W \partial U D_g\|_F^2 \\
&\qquad + \sum_g \|V_g^* + \partial V_g\|_{2,0,\Lambda} \\
&\quad \geq \|U^* - F\|_F^2 + \frac{\mu}{2} \sum_g \|V_g^* - WU^* D_g\|_F^2 \\
&\qquad + \sum_g \|V_g^* + \partial V_g\|_{2,0,\Lambda_g} \\
&\qquad + \mu \sum_g \langle V_g^* - WU^* D_g, \partial V_g\rangle
\end{aligned}$$

The inequality in the last line is obtained using (11) and the non-negativity of the Frobenius norm. Splitting the last two terms in this inequality with respect to index sets Γ_0 and Γ_1, using (12), and noting that $\|(V^* + \partial V)_{\Gamma_1}\|_{2,0,\Lambda} = \|V_{\Gamma_1}^*\|_{2,0,\Lambda}$ when $|\partial V|$ is small enough, we have

$$\begin{aligned}
&L_\mu(U^* + \partial U, V^* + \partial V) \\
&\quad \geq \|U^* - F\|_F^2 + \frac{\mu}{2} \sum_g \|V_g^* - WU^* D_g\|_F^2 \\
&\qquad + \sum_g \|V_{g,\Gamma_1}^*\|_{2,0,\Lambda_{g,\Gamma_1}} + \sum_g \|\partial V_{g,\Gamma_0}\|_{2,0,\Lambda_{g,\Gamma_0}} \\
&\qquad - \mu \sum_g \langle (WU^* D_g)_{\Gamma_0}, \partial V_{g,\Gamma_0}\rangle \\
&\quad = L_\mu(U^*, V^*) + \sum_g \|\partial V_{g,\Gamma_0}\|_{2,0,\Lambda_{g,\Gamma_0}} \\
&\qquad - \mu \sum_g \langle (WU^* D_g)_{\Gamma_0}, \partial V_{g,\Gamma_0}\rangle.
\end{aligned}$$

For a small enough $\|\partial V\|$, we have

$$\mu \|\partial V_{g,i}\|_F \|(WU^* D_g)_i\|_F \leq \Lambda_i \tag{13}$$

for $i \in \Gamma_0$, and using Cauchy-Schwarz inequality, we have

$$\sum_g \|\partial V_{g,i}\|_{2,0,\Lambda_{g,i}} - \mu \sum_g \langle (WU^* D_g)_i, \partial V_{g,i}\rangle \geq 0$$

and hence

$$L_\mu(U^* + \partial U, V^* + \partial V) \geq L_\mu(U^*, V^*).$$

□

We next derive the convergence results for the PD method, which generalize the results from [2]. We first establish the first-order necessary and sufficient optimality condition for problem (1).

Theorem 2. *Assume that* (U^*, V^*) *is a local minimizer of problem* (1). *Let* $J = \{1, \ldots, m\}$, $J_g^* = \{j \in J : \|(V_g^*)_j\|_2 \neq 0\}$ *and* $\bar{J}_g^* = J \setminus J_g^*$. *Then, there exists* Z^* *satisfying*

$$\begin{aligned} &2(U^* - F) + \textstyle\sum_g W^\top Z_g^* D_g^\top = 0, \\ &(Z_g^*)_{J_g^*} = 0, \;\; \textit{for } g = 1, 2, \ldots, G. \end{aligned} \tag{14}$$

Proof. It is not hard to observe that (U^*, V^*) is a local minimizer of problem (1) if and only if (U^*, V^*) is a local minimizer of the following problem

$$\begin{array}{ll} \min & \|U - F\|_F^2 \\ \text{s.t.} & (WUD_g)_{\bar{J}_g^*} = 0, \;\; \text{for } g = 1, \ldots, G. \end{array} \tag{15}$$

Then we have the Lagrangian function of (15)

$$L(U, V, Z) = \|U - F\|_F^2 + \sum_g \langle Z_g, WUD_g - V_g \rangle$$

where $Z = \{Z_1, Z_2, \ldots, Z_g\}$ and $(Z_g)_{J_g} = 0$. According to Proposition 3.1.1 in [3], we see that the conclusion holds. □

Theorem 3. *Let* (U^*, V^*) *be a feasible point of problem* (1), $J = \{1, \ldots, m\}$ *and* $J_g^* = \{j \in J : \|(V_g^*)_j\|_2 \neq 0\}$. *If for such* $\{J_g^*\}$ *there exists some* Z^* *such that* (14) *holds, then* (U^*, V^*) *is a local minimizer of problem* (1).

Proof. If for $\{J_g^*\}$ there exists some Z^* such that (14) holds, by Proposition 3.4.1 of [3] we know that (U^*, V^*) is a minimizer of problem (15) with $\bar{J}_g^* = J \setminus J_g^*$. Also, we observe that any point is a local minimizer of problem (1) if and only if it is a local minimizer of problem (15). It then implies that (U^*, V^*) is a local minimizer of (1). □

Theorem 4. *Let* $\{(U^k, V^k)\}$ *be the sequence generated by the PD method and* (U^k, V^k) *is the local minimizer of* $L_{\mu_k}(U, V)$ *obtained by the BCD method. Then, the following statements hold:*

(a) The sequence $\{(U^k, V^k)\}$ *is bounded;*

(b) Suppose (U^*, V^*) *is an accumulation point of* $\{(U^k, V^k)\}$. *Then,* (U^*, V^*) *is a feasible point of problem* (1).

(c) Let (U^*, V^*) *be as defined above. Suppose that* $\{(U^k, V^k)\}_{k \in K} \to (U^*, V^*)$ *for some index subsequence* K, *then* $\{\Omega^k\}_{k \in K}$, *with* $\Omega^k = \{\Omega_1^k, \ldots, \Omega_G^k\}$ *and* $\Omega_g^k = \mu_k(WU^kD_g - V_g^k)$, *is bounded. Moreover,* (U^*, V^*) *is a local minimizer of problem* (1).

Proof. In view of (3) and the safeguarding step with Υ in Algorithm 1, one can observe that

$$\begin{aligned} \|U^k - F\|_F^2 + \frac{\mu_k}{2} \sum_g \|WU^kD_g - V_g^k\|_F^2 + \sum_g \|V_g\|_{2,0,\Lambda_g} \\ = L_{\mu_k}(U^k, V^k) \leq \Upsilon, \quad \forall k. \end{aligned} \tag{16}$$

This immediately implies that $\|U^k - F\|_F^2 \leq \Upsilon$ and hence $\{U^k\}$ is bounded. Moreover, we can obtain from (16) that

$$\|WU^k D_g - V_g^k\|_F^2 \leq 2\Upsilon/\mu_k, \tag{17}$$

which, together with the boundedness of $\{U^k\}$, yields the boundedness of $\{V^k\}$. Therefore, statement (a) follows.

To show the validity of statement (b), since (U^*, V^*) is an accumulation point of $\{(U_k, V_k)\}$, there exists a subsequence $\{(U_k, V_k)\}_{k\in\mathcal{S}} \to (U^*, V^*)$. Then taking limits on both sides of (17) as $k \in \mathcal{S} \to \infty$, and using the fact that $\mu_k \to \infty$ as $k \to \infty$, we see that $\|WU^* D_g - V_g^*\|_F^2 = 0$. Thus, the conclusion holds immediately.

Next we show that statement (c) holds. We can easily observe that Ω^k is bounded from (17). Then we can let Ω^* be an accumulation point of $\{\Omega^k\}_{k\in K}$. By passing a subsequence if necessary, we can assume that $\Omega^k \to \Omega^*$ as $k \in K \to \infty$. Since (U_k, V_k) is the local minimizer of $L_{\mu_k}(U, V)$, by using the definition of Ω^k and from (11), we have

$$2(U^k - F) + \sum_g W^\top \Omega_g^k D_g^\top = 0. \tag{18}$$

Taking limits on both sides of (18), we see that the first relation of (14) holds with $Z^* = \Omega^*$.

Now let $J_g^* = \{j \in J : \|(V_g^*)_j\|_2 \neq 0\}$, $\bar{J}_g^* = J \setminus J_g^*$. Based on the definition of J_g^* and (12), we can observe that $(WU^k D_g)_{J_g^*} = (V_g^k)_{J_g^*}$ when $k \in K$ is sufficiently large. Hence, $(\Omega_g^k)_{J_g^*} = 0$ for sufficiently large $k \in K$. Thus, $(\Omega_g^*)_{J_g^*} = 0$. This together with the definitions of J_g^* and $\bar{J}_g^*$ implies that Z^* also satisfies the second relation of (14). It then follows from Theorem 3 that (U^*, V^*) is a local minimizer of (1). □

References

1. Zhang Y, Dong B, Lu Z. ℓ_0 Minimization for wavelet frame based image restoration. Mathematics of Computation. 2013;82:995–1015.

2. Lu Z, Zhang Y. Sparse approximation via penalty decomposition methods. SIAM Journal on Optimization. 2013;23(4):2448–2478.

3. Bertsekas DP. Nonlinear Programming. 2nd ed. Athena Scientific; 1999.
